# Government Initiative in Brazilian Public Health: A Technology Transfer Analysis

**DOI:** 10.3390/ijerph16173012

**Published:** 2019-08-21

**Authors:** Myller Augusto Santos Gomes, João Luiz Kovaleski, Regina Negri Pagani, Gilberto Zammar

**Affiliations:** 1 Department of Administration, Midwest University of Paraná. PR 153, KM 07, Irati 84500-000, Brazil; 2 Department of Industrial Engineering, Federal University of Technology-Paraná, Ponta Grossa 84016-210, Brazil

**Keywords:** technology transfer, public health, healthcare issues in technology, technology management

## Abstract

The objective of this research is to analyze the transfer of technology developed by the commercial and industrial compensation policy of the initiative of the Plan of Expansion of Radiotherapy of the Ministry of Health. The data of the organization subsidized by the Plan of Expansion of Radiotherapy were obtained through research documentary, interviews with professionals and participant observation. The methodological strategy consisted of a case study supported by a cross-sectional study, the internal environment was analyzed with variables found in the literature, allowing its comprehension in a certain hospital situation. The results revealed that the initiatives of introduction of radiotherapy equipment by the Expansion Plan suffered numerous confrontations within the contextual and organizational circumstances that affected its development and effectiveness. Given the struggles of the Expansion Plan at the tactical and operational levels of decision-making, there are challenges that require responses from organizations involved in the process to implement the trade agreement established by the compensation policy, with the initial stage being highlighted as a technology transfer process. Due to management skills and expertise, it gives you the paradigm status to be followed based on the relevant dimensions and indicators of the technology transfer analysis.

## 1. Introduction

Numerous countries have been facing a double challenge arising from two sets of changes caused by the modernization process: Social restructuring and economic conditions, both of which seem imperative to enable them to participate in an international economy increasingly more global in terms of technology transfer (TT), [1,2].

Emerging from public policies, government programs are State-sponsored initiatives in segments regarded as strategic areas, like security, healthcare, education, economy, and agriculture, with the purpose of ensuring citizenship rights through the access to public services.

The legislation on public procurement acts as a mechanism to make government action dynamic, establishing specific rules to ensure the practice’s effectiveness. Initially and informally, offset policies in Brazil, be they commercial, industrial, or technological, originated in the armed forces with the search for technologically-advanced military equipment abroad. As the offset, commodities and other means were provided as payment, in addition to the requirement of TT.

As the practice became more modern, segments of the State other than the military started to employ offset policies to acquire technologies in an effort to improve the efficiency of products and services. As a result of this practice, State policies have begun to promote another phenomenon: TT [3,4].

The TT processes in offset policies are complex due to populational divergences. Cultural aspects, industry attributes, and work organization are elements that may determine whether an initiative succeeds or fails [2]. In this perspective, the concern with technology’s adaptability to environments different than the original promotes essential discussions regarding aspects that are critical to obtaining satisfying results [5].

In this context, this paper analyzes the TT and the improvements in radiotherapy treatments in a case promoted by the Radiotherapy Expansion Plan, with the purpose of understanding the TT elements that affected the implementation and development of the solution. For this, a systematic literature review was developed capable of deriving elements that can be analyzed in the empirical context, thus extolling the relevance of this paper. This understanding could, in theory, lead to a means of analysis in other environments undergoing the actions of the Expansion Plan, thus developing an adaptation process [5,6,7,8,9].

## 2. Methodology

The systematic literature review used the methodology Methodi Ordinatio, working with materials already published and found through a search in the databases. The research used keyword combinations without time restrictions due to the limited phenomenon of the study. The results included published articles, which were then analyzed to see if they fulfilled the research question and the study phenomenon. The intention of the systematic literature review is to find variables that can be analyzed in a given context, taking into consideration the research question. Developed by [10,11], was employed to rank papers by their scientific relevance, taking into account the variables impact factor, year of publication, and the number of citations. The phases of Methodi Ordinatio are:

Phase 1—Establishing the intention of research: ‘The relationship between TT and offset policy’.

Phase 2—Definition of databases: ‘ScienceDirect, Web of Science and Scopus’.

Phase 3—Definition of keywords combinations.

**Figure 1:**
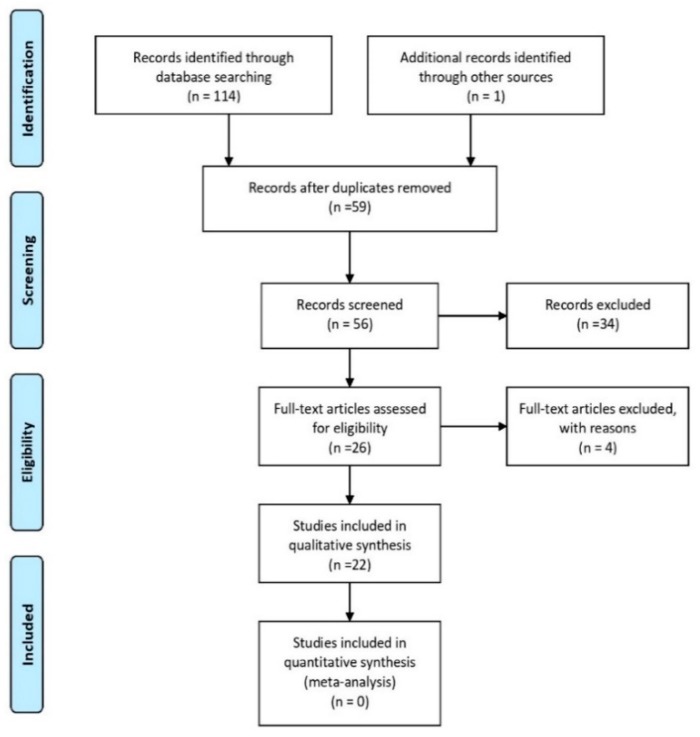
Flow chart of studies included in the review.

Phase 4—Final search in the databases and gross results are described in Figure 1.

Phase 5—Filtering procedures: Were used to eliminate duplicated papers; papers not related to the theme.

Phase 6—Identifying impact factor, year and number of citations: The metrics of the papers (impact factor) were obtained from the Thomson’s Reuters/Clarivate Analytics website. If the paper does not use JCR, the next metric will be to search on the Scopus website, following this order of preference: CiteScore, SJR (Scimago) ou SNIP. The number of citations was obtained from Google Scholar. This information metrics and number of citations along with the year of publication, is necessary to calculate the InOrdinatio.

Phase 7—Ranking the papers using the InOrdinatio: In order to rank the papers according to their scientific relevance, the index of ordination—the InOrdinatio.

Equation (1)—is applied, using an electronic spreadsheet.

InOrdinatio = (IF/1000) + α × [10 − (ResearchYear − PublishYear)] + (Ci)(1)

The Factors of the equation are: IF (impact factor); α (alpha value, ranging from one to ten, to be defined by the researchers according to the importance of the newness of the theme; for this study, the value of α was defined to be ten, since the theme is the object of the study); ResearchYear (year in which the research was developed); PublishYear (year in which the paper was published) and Ci (number of times the paper has been cited).

Phase 8—Finding the full papers: Papers were collected and stored using the reference manager Zotero®.

Phase 9—Reading and systematizing analysis of the papers: Bibliometric mapping and content analysis were used to evaluate the papers.

The purpose of this process was to find common elements so as to devise a model able to observe how the TT phenomenon takes place in public health. Elements related to general characteristics and organizational conditions in a determined workplace received a particular focus. Establishing indicators based on these elements and carrying out an in-depth analysis points out the positive and negative overflows of the TT process.

Therefore, the definition of dimensions and indicators occurs in agreement with the initial research question and the systematic literature review, meeting the theoretical and methodological approach selected for the study. The goal is to find the knowledge contained in the workplace, allowing for an in-depth analysis. This research found dimensions and indicators suitable for the context of implementing the linear particle accelerator in hospitals.

Documentary research, informal interviews, and participant observation were necessary to understand the context of analysis based on the dimensions and indicators proposed. The variables describe the general and organizational characteristics of the workplace studied. Table 1 displays the dimensions.

## 3. Radiotherapy Expansion Plan

Established by the Ordinance No. 931, of 10 May 2012 of the Ministry of Health, the radiotherapy expansion plan of the Brazilian Unified Health System (*SUS—Sistema Único de Saúde*), represents the largest worldwide investment in the acquisition of linear accelerators, with the aim of expanding the treatment network in the therapeutic modality of radiotherapy. This plan was executed with the use of the mechanisms of the offset policy, to the commercial purchase contract.

Composed of 80 solutions in radiotherapy, it can be comprehended as the creation and expansion of radiotherapy centers. For the process of operationalization of the plan, the Ministry of Health divided the process into two stages, as explained in, 1st stage, acquisition of the basic architectural and executive projects; services to support the fiscalization and supervision of the stage execution; equipments and the effective technological compensation, 2nd stage, contracting of services to the building of the planned structure, according to a basic project and execution of the previous stage, to receive the acquired equipment.

It deals with the acquisition of linear accelerators, an equipment used in the external radiotherapy modality, that allows the professionals to control the intensity and directionality of the ionizing rays. The linear accelerators are a consistent evolution of modern radiotherapy, which seeks to develop reliable and controllable means for the radiation process, being considered the greatest reference in radiotherapy treatments. However, the determinant factor for the use of this equipment lies in the anatomical precise location of the malignant cells.

In addition to the acquisition of the equipment, the plan in the commercial agreement provides for a technology compensation agreement, which aims to build the industrial environment of linear accelerators, qualification of local suppliers for the production of components, accessories and software. In addition to the qualification of professionals, the Varian Medical Systems Co. (Palo Alto, Unites States of America) must qualify suppliers selected by it; and establish agreements with scientific and technological institutions for the technology transfer focused on the software development. For the partnership with the scientific institutions selected in the previous stage, the selected company must keep a place to train engineers, physicists, technicians and related professionals focused on the qualification about linear accelerators.

There is a great concern about the final result, since the synchronization between stages is fundamental to the effectiveness of the plan. There are different institutions and people involved, such as the Ministry of Health, Varian Medical Systems Co., State Secretaries of Health, the hospitals covered by the plan and the contractors responsible by the planned work, as well as the scientific and technological institutions participating in the public call for technology transfer. Consequently, the institutional efforts are fundamental for the solution in radiotherapy to be made available. However currently, the evolution scenario of the plan is slow since 2012. According to data collected from June 2017, the current status of the plan was presented: Only three solutions were delivered; six are in execution; one project is halted; six in the bidding process; one in elaboration of the reference term; eight with executive projects under analysis; three executive projects in the Varian company; 26 with basic architecture and engineering projects in the reanalysis processes; one basic design of architecture and engineering in analysis; 21 projects were suspended by determination of the executive committee and four projects were excluded by the managing committee.

Concerning the three solutions delivered, the current situation is as follows: The first solution was delivered in November 2016 in the State of Paraíba; the second solution delivered in May 2017 in the State of Bahia; and the third solution in June 2017 in the State of Paraná. So, compared to the first schedule, it is clear that the inefficiency of the contextual analysis represents the main problem or obstacle in the insertion of the solutions in hospitals accredited for the oncological treatment.

## 4. Results and Discussion about TT in the Organization Selected by Radiotherapy Expansion Plan

**Figure 2:**
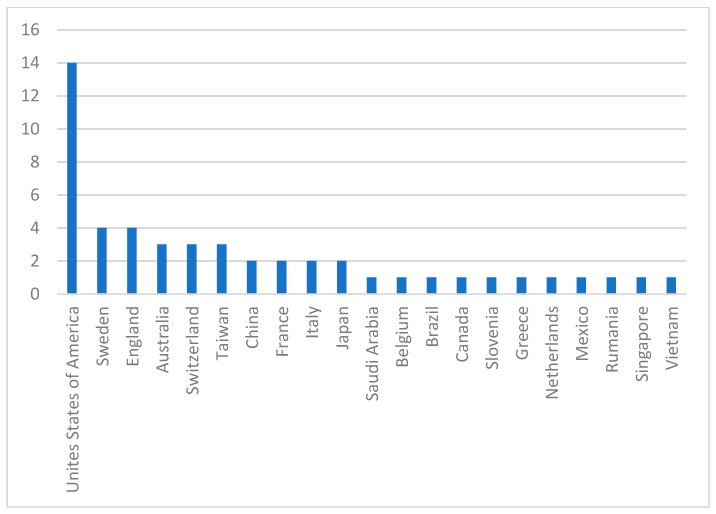
Nations that promote research on technology transfer in offset policies. Source: Research Data.

**Figure 3:**
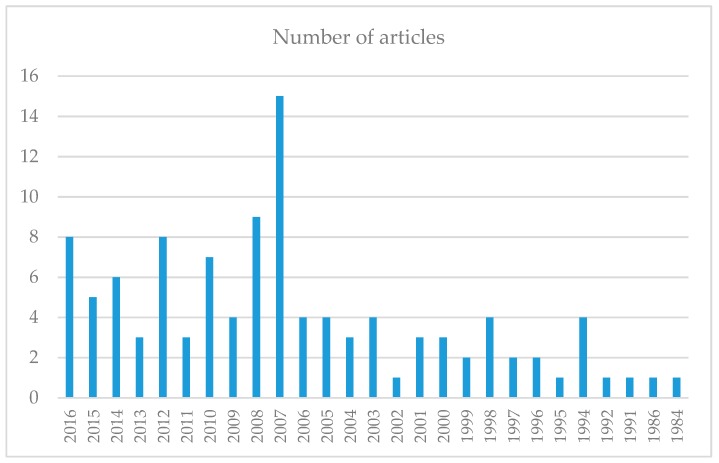
Respective years of publication of articles. Period: “AllYears”. Source: Research Data.

Systematic literature review data, newborn licensing, countries that publish on the subject, what phases of publication have occurred, are the ones that most reveal that the highest number of citations are shown in Figure 2 and Figure 3 and Table 2.

Figure 3 presents the years of the respective publications considering the articles that answer the research question.

Thus, it was possible to identify the most cited authors and the ten most relevant works according to the methodology, as follows.

The following Table 3 highlights the journals that had the highest number of citations involving the combined keywords.

The main articles cited in the systematic literature review are described in Table 4. These articles have a common procedure, a qualitative approach and consider offset policies.

### Evidence

This section describes the empirical research that has been conducted in recent years in a hospital that has received improvements proposed by the offset policy, with the intention of testing the dimensions and indicators proposed by the systematic literature review.

General characteristics: The organization selected by the Radiotherapy Expansion Plan is located in Curitiba, State of Paraná. It is regarded as a model of excellence in oncology treatments in the South of Brazil. Founded in 1972 by civil society initiatives, the organization at hand has specialized over time in oncology and has actively participated in the SUS since its creation.

The radiotherapy service at the organization, since its creation, has been prone to expanding its operations, with easy access and high patient flow in an area of 677.01 m^2^. The area selected for the implementation of the linear accelerator stated in the plan is of 188.51 m^2^.

The organization features a technological park that contains equipment able to conduct radiotherapy treatment in its several modalities. Currently, it has three linear accelerators, one telecobalt machine, high dose-rate brachytherapy, and now the linear accelerator acquired via the Radiotherapy Expansion Plan, including built-in software, which comprises technologies that act in the machinery’s functionality that require profound understanding by the users so as to correctly operate it [12,14].

Acquired via a process of international business, the linear accelerator has a high aggregated value due to the built-in technologies related to its operation. For developing countries like Brazil, it is still necessary to purchase from developed countries and initiate stages of technology transfer with adaptations aiming to bring out the full potential of the social and economic value of the implementation process [15].

During the participant observation and informal interviews with professionals of the radiotherapy sector, it was possible to identify that, within the initial selection parameters of the Radiotherapy Expansion Plan, the organization had not been chosen. However, realizing the difficulties of implementing and developing the project, the Ministry of Health proposed several additions to the program as an alternative to solve problems. Organizations accredited in oncology by the Ministry of Health were invited to facilitate the absorption of new equipment so as not to negatively impact the technology transfer process, seeing that their management has the capabilities and expertise required [16,17]. The existence of those elements allows for the licensing of operations involving the linear accelerator equipment.

**Figure 4:**
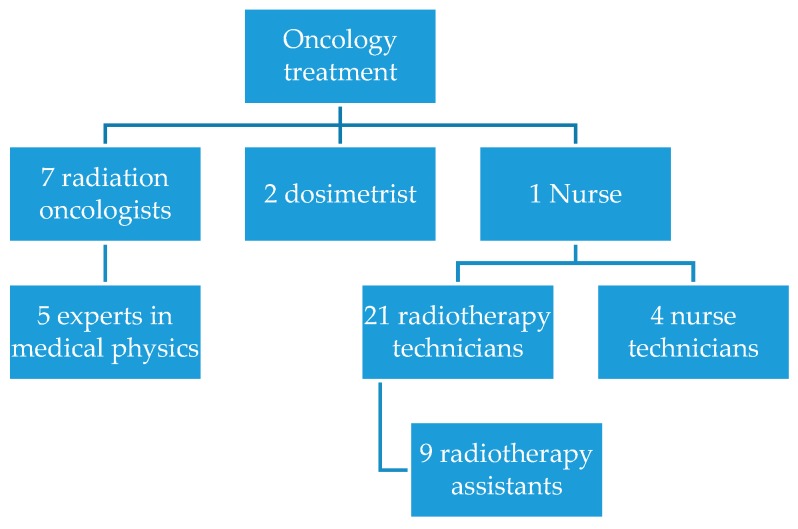
Number of professionals in the radiotherapy sector.

Organizational characteristics: The organization has approximately 1038 employees in its entire organizational environment. Figure 4 displays the composition of the staff in the radiotherapy sector, in terms of numbers and functions.

This staff team is responsible for every radiotherapy treatment conducted by the organization, a monthly average of 20,230 radiotherapy applications.

It presents a mechanistic hierarchical structure in virtue of its departmentalization. However, the relations between departments (sectors) occur harmoniously, creating a favorable organizational climate [18]. The elements that enable said harmony are related to their customers (patients), who undergo a delicate process. The organizational climate becomes different in an effort to sympathize with the patients’ condition.

The hiring process is conducted by the organization itself. Its webpage presents the menu “work with us”, indicating the availability of openings regardless of function. In this menu, it is possible to attach résumés and to view the abilities and competencies required to enter the selection process for an available opening. The selection and recruiting stages are conducted by the relevant sector and the personnel management sector.

The main activities conducted by the organization are oncology-related specialties with emphasis on clinical analysis, pathological anatomy, anesthesiology, head and neck, cardiology, abdominal surgery, plastic surgery, vascular surgery, palliative care, endoscopy, gynecology and breast care, neurosurgery, ophthalmology, clinical oncology, orthopedics, pediatrics, skin and melanoma, radiodiagnosis, radiotherapy, intensive therapy, thorax, urology, and oral and maxillofacial.

Organizational aspects of the work process: The activities start at 7 a.m. and end at 10 p.m. divided into three work shifts. In the division of labor in the radiotherapy sector, the 8 a.m.–4 p.m. period is reserved for previously-scheduled medical appointments. The radiotherapy treatments are conducted during the entire 15-h period, from 7 a.m. to 10 p.m. On average, each session lasts no more than 12 min.

Due to the particularity of the context, the employees’ workloads vary; the radiation oncologists and one medical physicist work 20 h weekly while four medical physicists work 40 h. The technicians and their assistants follow a daily schedule of 4 h and 48 min.

**Figure 5:**
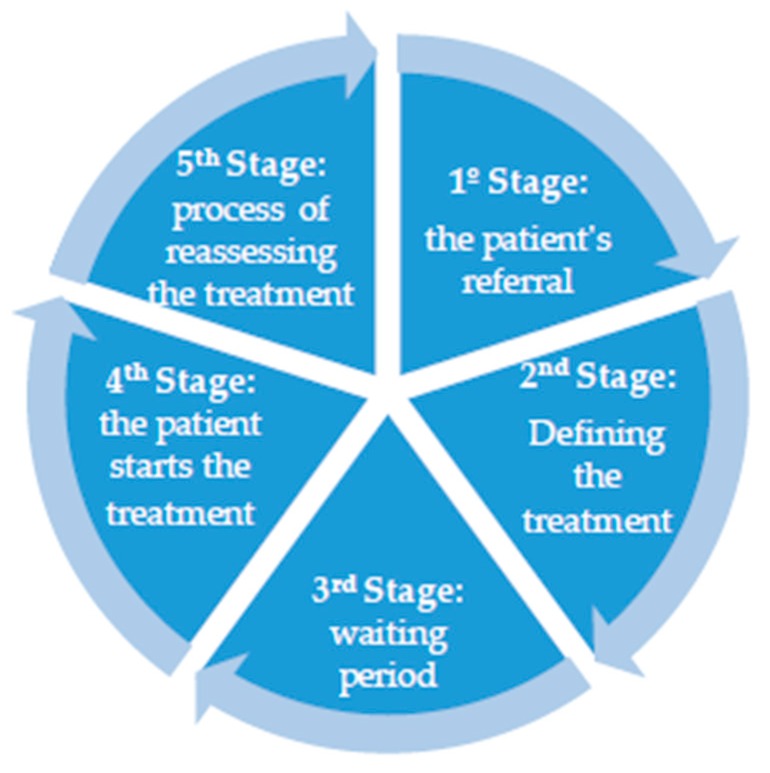
Flowchart of patient care in the radiotherapy sector.

The productive system of patient care in the organization works systematically, managing the care of new patients, supporting patients undergoing treatment, and following up on patients who have been discharged from the proposed radiotherapy treatment. This allows the organization to pace the existent demand, monitor the use of the technological park, and receive improvements promoted by the SUS without affecting the patient care system, which is displayed in stages by the following flowchart (Figure 5).

1st stage: It comprises the patient’s referral, in which appointments and records are made. It functions from 7 a.m. to 4 p.m., and the appointments are scheduled between 8 a.m. and 4 p.m.

2nd stage: The radiation oncologist conducts the appointment, assessing the patient and defining the treatment. In some cases, a three-dimensional (3D) planning with a tomography or a bi-dimensional (2D) planning with simulators is required. The planning allows for the use of several radiation fields in the assessment of the radiation dose for the organs at risk, in order to improve the accuracy in the radiation dose delivered to the target organ [18,19].

3rd stage: The patient undergoes a waiting period, which is necessary to organize the current demand in the organization. Usually, 3D planning requires 15 days on average, whereas 2D planning requires eight days. Moreover, the medical physicist needs a specific time period to make the necessary calculations to initiate the patient’s treatment.

4th stage: The patient starts the treatment through linear accelerators or the telecobalt machine.

5th stage: The radiation oncologist conducts the process of reassessing the treatment after its conclusion; the resulting reports are registered into the patient’s records.

Following the organization’s procedure for patient care, the management of tasks and activities takes place individually in each stage, which means that every patient has access to their medical record and the relevant information concerning the treatment.

The ability of the radiotherapy sector to care for patients is defined by the rate in which they arrive and enter the patient care process. These metrics depend on the availability of human resources, as well as the demand for scheduling initial appointments [19]. The seven radiation oncologists conduct 48 appointments a week, on average, disregarding return visits and reassessments.

**Figure 6:**
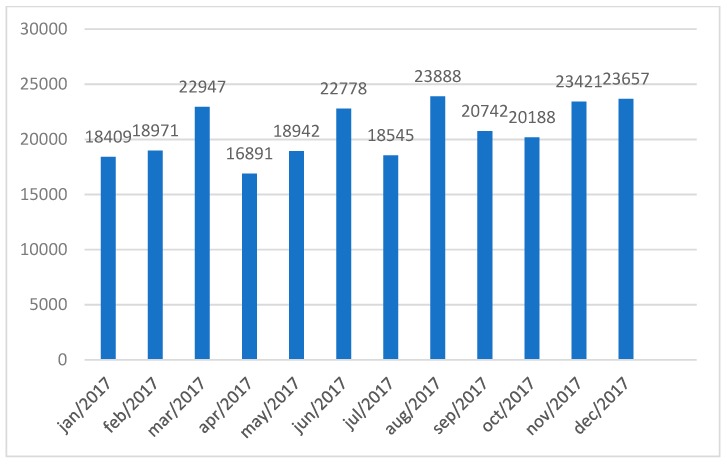
Applications in 2017. Source: Research data.

Until December 2017, the average of radiotherapy applications per month was of 20,782, 91% of which were arranged via SUS. Figure 6 presents the number of applications conducted by the radiotherapy sector, taking both external and internal procedures into account.

In June 2017 the linear accelerator acquired via the Radiotherapy Expansion Plan was activated, increasing the daily capacity of treatments. On average, one linear accelerator can treat 270 patients a day in sessions of at most 12 min. This applies to three linear accelerators and one undergoing maintenance. Installing the linear accelerator acquired via the Radiotherapy Expansion Plan has increased the organization’s capacity by 90 patients per day.

Technical factors: The organization has a technological park of radiotherapy that features one cobalt therapy machine and four linear accelerators of the brand Varian Medical Systems, models 2100, 600CD0, 600CD3, and Clinac XC, with the latter being the one acquired via the plan.

The technological park also contains the Acuity simulator, the GE simulator with an ADW40 workstation dedicated to radiotherapy, beta radiotherapy with Strontium-90 plaques for surface treatment, plaques for dermatological care, plaques for ophthalmological care, high dose-rate brachytherapy, and planning and management software.

Employing models produced by a single manufacturer facilitates the maintenance procedures, especially for the existent linear accelerators in the aspect of exchangeability of parts, in addition to establishing procedures of preventive maintenance conducted by the engineering and maintenance sector of the organization [18,20,21]. The institutional relationship between Varian and the organization were consolidated before the Radiotherapy Expansion Plan, which promotes promptness during maintenance in meeting specific needs, such as restocking components that require replacements as the equipment is used [21].

This section discusses the general characteristics and the organizational conditions of the analyzed workplace, investigating how the linear accelerator was introduced and its contribution to the organization.

Developing an analysis of the indicators proposed, the organization benefited by the Radiotherapy Expansion Plan presented itself as an alternative to the problems of selection and credit found in other hospital units. Moreover, it has further advantages in the management process; in virtue of its status of a non-profit organization, it externalizes its institutional actions [22]. With clarity in its mission and systematization regarding the origin of patients, the organization provides, through its infrastructure, professionals and partnerships to fulfill its mission and goals.

Particularly for the radiotherapy sector, the existence of the technological park reveals the diversity of technological resources dedicated to radiotherapy. Aligned with the goal of the Radiotherapy Expansion Plan, the organization met every prerequisite for expanding its current services. Nevertheless, the clinical staff of the radiotherapy sector is divided: During the participant observation, talking to the professionals revealed that a few approve those acquisitions, seeing that they improve the equipment availability for the SUS. Others, however, report that this type of technology for cancer treatment is obsolete compared to the one already employed in the technological park and that an ideal alternative would be to increase the diversity of specialties and develop surgery capability. In their point-of-view, the government ended up creating 80 new problems for public health.

Given these results, it is evident that the growing demand of new cases of cancer suggests preventive measures to combat it directly. However, most new cases arise, on average, between the initial and intermediary stages, which requires the hospital structure to have treatments capable of mitigating the unavoidable impacts caused by the disease. In a context of historical obsolescence of investments in equipment, the Plan presents itself as an attempt, jointly with other measures, to strengthen the SUS in the oncology department.

Another aspect is the installation of the linear accelerator. In the organization, due to its competencies and abilities, the introduction of the equipment to the treatment process occurred efficiently and smoothly. The clinical staff was essential, due to their already steadfast experience of acting with this type of equipment over time, which removed the need for training personnel during the process [23].

It is a fact that the capacity of radiotherapy applications increased considerably with the arrival of the linear accelerator, considering the demand for appointments and treatments in the organization. The pretension to acquire equipment that would diversify specialties is understandable, but having the technological infrastructure to increase the volume of treatments evidently improves the care provided [23].

The next point of analysis approached the organizational conditions in the work environment and revealed that the productive system of patient care is systematized into stages, which allows for dynamic management considering the variables of the process. Stages 2 and 5 are seen as critical. Stage 2 is responsible for the planning process involving the radiation oncologist, the medical physicist, and the dosimetrist. The time required for the communication between these professionals is around 15 days, to effectively start the treatment. Stage 5 involves the reassessment process, post-treatment.

The cruciality of both stages lies in the execution time and in the patient’s waiting. Increasing the amount of employees is recommended so as to decrease the waiting period between the first appointment and the treatment. The current system, jointly with procedures adopted and validated based on practice, may contribute to the TT process, stimulating the employees to provide subsidies that can be employed in a future proposal of an upgrade for the built-in information systems, scheduled in the technological offset.

Disregarding the first stage of the Radiotherapy Expansion Plan as a TT process obfuscates the perception of existence and contribution. This context requires a reflection about the wealth of information and subsidies from different origins within the plan, which allows for the discovery of potentialities that can be introduced to linear accelerators and optimize the process of radiotherapy treatment [8,9,16]. Observing management practices of infrastructure and patients in the organization serves as an example for other hospital units, considering that its organizational characteristics are effective and its institutional actions are clear and objective, in addition to the demand that it absorbs in its geographic, demographic, cultural, and anthropological context.

Despite the divergent contexts to which the solutions proposed by the plan are introduced, comprehending the external and internal environments of each situation is essential, seeing that these variables may lead to overflows. The involved parts must find adaptations so as to optimize the reach and the care provided by the SUS.

## 5. Conclusions

The purpose of this paper, analyzing TT in the introduction process of the linear accelerator to the hospital environment, was achieved through the systematic literature review and the construction of the analysis model based on dimensions and indicators, which illustrate the context of the workplace. The study identified that most difficulties in the operationalization of the Radiotherapy Expansion Plan in Brazil involve the internal environments in different contexts. The situation defined for the case study showed, explicitly, that the introduction process of the linear accelerator studied in the State of Paraná succeeded due to the particular set of general and organizational characteristics.

The TT processes included in the offset policy seem to be in the background at first, as the acquisition and the commercial relationship become the main focus. However, the positive and effective overflow of TT may contribute to the technological development of the receiving organizations, enable economic growth, and intensify international relationships centered on commercialization, TT, product research and development, and know-how creation. On the other hand, the negative overflow involves the failure to complete the TT, not upgrading the equipment and lacking effectiveness in the optimization and fulfillment of the commercial agreement. Therefore, the ideal procedure is to complete the TT first, before the equipment arrives. That way, the improvements may occur beforehand, which does not limit the TT to only being an offset of the commercial agreement.

The process behaves linearly, transferring the technology contained in the equipment acquired. The study about the offset policy in public health revealed the complexity of the activities that rely on inter-organizational relations, in which structured activities and centralized decision-making process make the procedures slow and unstable. However, considering the process of installing new equipment as TT and reflecting on different contexts and environments in terms of adaptations may represent strategic and operational gains for the institutions involved.

This experiment is limited to hospitals that represent a high standard in cancer treatments which have been considerably improved via the radiotherapy expansion plan. Other initiatives have attempted to accredit the hospital in cancer treatments but, unfortunately, have not panned out. Other hospitals that meet the same high standards in oncology in Brazil were not considered due to their low participation rate in the SUS.

This analysis model was applied to a hospital, examining 13 indicators of the internal environment that influence the performance in TT and oncology treatments. The proposal is to broaden the sample and conduct analyses in other hospitals, under different contexts and organizational characteristics, so that the proposed model can evolve. The actions and recommendations for public health authorities in Brazil are as follows: (1) The application of the model in a larger and more complex hospital in oncology treatments linked to the SUS; (2) the application of the model of TT analysis in different hospitals, comparing the results found in each context to ascertain the difference between each work process in oncology treatments; (3) conduct a comparison between large and small hospitals and (4) the application of the analysis model to a considerable number of hospitals in different countries, comparing developed and developing countries in terms of TT related to linear accelerators and other methods of oncology treatments. Studies based on the analysis model and the experience of the hospital with high standards may be easier to understand for both interviewers and interviewees, possibly leading to different findings.

## Figures and Tables

**Table 1:** Dimensions and indicators of analysis.

Dimensions	Indicators	Author
General characteristics	Work process	[12]
	Materials required for the process	[13]
	Recommendations adopted	[6]
Organizational characteristics	Staff	[8]
	Hierarchical structure	[1,2]
	Means of hiring personnel	[14,15]
	Main activities	[16,17]
Organizational aspects of the work process	Schedule and division of labor	[5,7,18]
	Work organization and structuring	[19,20]
	Control over task fulfillment	[3,21]
	Capacity of daily treatments.	[9,15]
Technical factors	Available equipment	[4]
	Maintenance procedures	[22]

Source: Research data.

**Table 2:** The most cited authors regarding technology transfer (TT) and offset policy *.

Authors	No. of Citations
1.Autken and Harrison (1999)	4628
2.Mencinger (2003)	380
3.Thurow (1997)	347
4.Maskus and Reidman (2004)	290
5.Bidaut and Cummings (1994)	214
6.Anderson (2005)	182
7.Vishwasrao (1994)	156
8.Muller (2007)	101
9.Branstetter and Saggi (2011)	92
10.Macpherson (2003)	53

Source: Research data.

**Table 3:** Main Journals and frequencies of publications.

Journal	Number of Articles
American Economic Review	5
Energy Policy	3
Research Policy	2
Technovation	2
Applied Ergonomics	1
Competition & Change	1
Economic Journal	1
International Journal of Radiation Oncology Biology Physics	1
Journal of Development Economics	1
Journal of International Economic Law	1
Journal of manufacturing technology management	1
Journal of technology transfer	1
Kyklos	1
SpringerPlus	1
Traivaller	1
World Development	1

Source: Research Data.

**Table 4:** Main studies analyzed.

Source	Purpose of the Study	Research Procedure
Autken and Harrison (1999)	Investigated the relationship of foreign direct investment with technological overflows in domestic companies in Venezuela.	Data from 4000 companies being joint venture and domestic companies in Venezuela.
Mencinger (2003)	Explores the relationship between FDI and economic growth in eight transition countries—EU candidates-in the period 1994–2001.	Statistical analysis of foreign direct investment and economic growth.
Thurow (1997)	Discusses the need for a new intellectual property rights system.	Comparative analysis of common and unusual features in intellectual property law systems.
Maskus and Reidman (2004)	Argues that the globalized IP regime will strongly affect prospects for technology transfer and competition in developing countries.	Answers from experts in intellectual property law.
Bidaut and Cummings (1994)	Analyzed the innovation processes occurring in several cross-industry technology partnerships.	Literature review on limitations in technological solutions.
Anderson (2005)	Analyzes rising income inequality in developing countries receiving foreign direct investment.	Developed a literature review and empirical study on income inequalities in relation to the hypotheses formulated by the theory.
Vishwasrao (1994)	Incorporates asymmetric information in a screening game where the innovating firm has the choices of licensing a new product at arm’s length to a foreign firm, exporting it.	Online research with experts.
Muller (2007)	Proposes two extra questions about clean development mechanisms and the promotion of sustainable development.	Research with experts.
Branstetter and Saggi (2011)	Develops a model for strengthening intellectual property rights.	Exploratory Approach on relevant experiences and the construction of a mathematical model.
Macpherson (2003)	Analyzes how compensation arrangements affecting US exporters.	Interviews with 48 export-intensive companies.

Source: Research data.
